# The Role of Perinatal Complications in Neurodevelopmental Outcomes of ART-Conceived Children: Prognostic Model for Brain Immaturity

**DOI:** 10.3390/biomedicines13102551

**Published:** 2025-10-20

**Authors:** Sevara Ilmuratova, Vyacheslav Lokshin, Zhanar Nurgaliyeva, Kаnatzhan Kеmelbekov, Gulshat Kulniyazova, Bibigul Abdykalykova, Roza Seisebayeva, Karlygash Zhubanysheva, Gulmira Altynbayeva, Gulnar Mukhambetova, Ainur Sadykova, Damir Marapov, Valeriya Nekhorosheva, Lyazat Manzhuova

**Affiliations:** 1 International Clinical Centre of Reproduction “PERSONA”, Almaty 050060, Kazakhstan; v_lokshin@persona-ivf.kz; 2 Department of Outpatient Pediatrics, School of Pediatrics, Kazakh National Medical University named after S.D.Asfendiyarov, Almaty 050012, Kazakhstan; nurgaliyeva.z@kaznmu.kz (Z.N.); seisebaeva_68@mail.ru (R.S.); 3 Department “Pediatrics-1”, JSC South Kazakhstan Medical Academy, Shymkent 486019, Kazakhstan; kanat-270184@mail.ru; 4 Department of Fundamental Medicine, Al-Farabi Kazakh National University, Almaty 050040, Kazakhstan; k_gulshat@mail.ru; 5 Department of Obstetrics and Gynecology, School of General Medicine No. 2, Kazakh National Medical University named after S.D.Asfendiyarov, Almaty 050012, Kazakhstan; abdykalykova.b@kaznmu.kz; 6 Department of Neonatology, Kazakh National Medical University named after S.D.Asfendiyarov, Almaty 050012, Kazakhstan; karlygash77@bk.ru; 7 Scientific Center of Pediatrics and Pediatric Surgery, Almaty 050012, Kazakhstan; altynbayevag22@gmail.com (G.A.); ljazat.manzhuova@mail.ru (L.M.); 8 Department of Nervous Diseases, Kazakh National Medical University named after S.D.Asfendiyarov, Almaty 050012, Kazakhstan; gulnar2311@mail.ru; 9 Department of Infectious and Tropical Diseases, School of General Medicine No. 2, Kazakh National Medical University named after S.D.Asfendiyarov, Almaty 050012, Kazakhstan; 10 Department of Public Health, Economics and Health Care Management, Kazan State Medical Academy, 420012 Kazan, Russia; damirov@list.ru; 11 Institute of Reproductive Medicine, Almaty 050012, Kazakhstan; nehorosheva1987@gmail.com

**Keywords:** assisted reproductive technologies, neurodevelopment, brain immaturity, prognostic model, perinatal complications

## Abstract

**Background/Objectives**: Since the first successful birth following assisted reproductive technologies (ART) several decades ago, the global population of ART-conceived children has surpassed 13 million, with over 40,000 born in Kazakhstan. Despite this growth, questions remain about their long-term neurological outcomes, with existing studies reporting inconsistent findings. This study aimed to assess psychomotor development and the prevalence of nervous system pathologies among ART-conceived children in Kazakhstan and to develop a prognostic model for identifying pathological neurodevelopmental conditions. **Methods**: We studied 252 children (120 conceived via ART and 132 controls) using clinical examination and medical history data. Brain immaturity predictors were identified by univariate and multivariate logistic regression. **Results**: ART-conceived children exhibited a higher incidence of neurosonographic signs of brain structure immaturity. However, multivariate analysis indicated that ART itself was not an independent risk factor. Instead, perinatal complications—including prematurity, multiple pregnancy, low birth weight, asphyxia, and intrauterine infections—explained the observed differences. The prognostic model highlighted prematurity and preconceptional progesterone therapy as significant predictors. Overall neurological development did not differ significantly between the groups. **Conclusions**: These findings underscore the importance of early identification of perinatal risk factors and targeted preventive interventions to mitigate adverse neurodevelopmental outcomes in ART-conceived children.

## 1. Introduction

Since the birth of the first child conceived by assisted reproductive technologies (ART), these methods have become an essential part of infertility treatment worldwide [1]. To date, more than 13 million children have been born following ART procedures [2], including over 40,000 in Kazakhstan [3]. This rapid expansion has raised increasing concern regarding the long-term health of ART-conceived offspring, particularly in relation to neurological and psychomotor development.

Current evidence remains conflicting. Several population-based cohort studies have reported higher rates of neurological morbidity in ART offspring compared with naturally conceived (NC) children, including increased prevalence of attention deficit hyperactivity disorder (ADHD), autism spectrum disorders (ASD), and cerebral palsy (CP) [4,5,6,7]. However, these associations often diminish after adjusting for confounding factors such as prematurity, multiple gestations, and low birth weight. While some studies, such as Hansen et al. [8], suggest a modest increase in intellectual disability among ART children—especially those born preterm—others indicate no significant differences in long-term mental health outcomes [9,10].

Cognitive and educational outcomes are also debated. Some investigations describe slightly delayed language and developmental milestones in early childhood, whereas others demonstrate comparable or even superior school performance in ART offspring compared to NC peers [11,12,13]. These discrepancies likely reflect the influence of perinatal complications, parental factors, and regional differences in ART protocols.

Biological mechanisms potentially underlying neurodevelopmental differences in ART offspring include epigenetic alterations induced by in vitro culture conditions, ovarian stimulation, and gamete or embryo cryopreservation [14,15,16]. Additionally, ART pregnancies are associated with higher risks of adverse perinatal outcomes such as preterm birth, intrauterine growth retardation (IUR), asphyxia, and infections, all of which may contribute to later neurological vulnerability [17,18,19,20,21,22,23,24,25,26]. Despite improvements in ART techniques, including single embryo transfer, concerns remain regarding long-term developmental trajectories.

In summary, the current literature suggests that ART itself may not constitute an independent risk factor for adverse neurodevelopment, but rather that the increased risk is mediated by perinatal and maternal factors. Importantly, most available data come from Europe, North America, and East Asia, whereas evidence from Central Asia is lacking.

The present study aims to evaluate the prevalence of neurological disorders among ART-conceived children in Kazakhstan and to identify associated perinatal and clinical risk factors.

## 2. Materials and Methods

This observational controlled study involved children conceived by ART from three leading reproductive centers in Kazakhstan (the International Clinical Center of Reproductology PERSONA, the Institute of Reproductive Medicine, and the ECOMED Clinic). The study was part of the scientific and technical project ИРН AP26100341. Inclusion criteria for the ART group were a successful ART program using in vitro fertilization (IVF) or Intracytoplasmic sperm injection (ICSI), with fresh and frozen-thawed embryo transfer, and childbirth between 2018 and 2023. The ART cohort included both single and multiple embryo transfers. Exclusion criteria included intrauterine insemination with sperm from the woman’s partner or a donor, as well as participation in programs that involve donor gametes or surrogacy. Comprehensive medical history data were collected for both groups, and all children underwent thorough examinations.

### 2.1. Definitions of Pathological Neurodevelopmental Conditions

We applied the following standardized diagnostic criteria:

- IUR: Defined as estimated fetal weight or birth weight below the 10th percentile for gestational age according to WHO guidelines.
- Birth trauma: Diagnosed according to ICD−10 codes P10–P15, including intracranial hemorrhage, brachial plexus injury, and skeletal fractures sustained during delivery.
- Nervous system damage: Included perinatal hypoxic–ischemic encephalopathy and other central nervous system injuries documented in neonatal medical records.
- Immaturity of brain structures on neurosonography (NSG): Identified by a pediatric neurologist and radiologist based on ultrasonographic markers, including persistence of germinal matrix, ventricular asymmetry, and delayed cortical maturation.
- Cognitive impairments: Defined as developmental delay or intellectual disability recorded in pediatric follow-up, corresponding to ICD−10 codes F70–F79.
- ADHD: Diagnosed by pediatric psychiatrists according to DSM−5 criteria and confirmed in medical history.
- ASD: Diagnosed by pediatric psychiatrists based on ICD−10 F84 codes and standardized behavioral assessment.
- Mental disorders: Included documented diagnoses of mood, anxiety, or behavioral disorders, classified under ICD−10 F30–F99 codes.

These criteria ensured methodological transparency and consistency with international diagnostic standards.

### 2.2. Statistical Analysis

Statistical analysis of the collected data was conducted utilizing the IBM SPSS Statistics software (version 26; SPSS, Inc., Chicago, IL, USA). To evaluate the normality of the sample distribution, the Kolmogorov–Smirnov test was employed. For the assessment of differences in variable values between the groups, multifield contingency tables were utilized in conjunction with Pearson’s χ^2^ test and Fisher’s exact test. Correlations among variables were examined using the odds ratio (OR). In instances where the occurrence frequency of a trait in one group was zero, the Haldane-Anscombe correction was applied for the calculation of the OR, utilizing an online calculator: available at: https://www.medcalc.org/calc/odds_ratio.php. Accessed 31 August 2025. Statistical significance was determined at a *p*-value threshold of less than 0.05. The establishment of a prognostic model aimed at evaluating the risk of a specific outcome was executed using the binary logistic regression method. This methodological selection was chiefly motivated by the dichotomous nature of the dependent variable (for example, the presence or absence of the pathological condition), while the independent variables comprised both categorical (nominal or ordinal) and quantitative characteristics. The logistic regression model calculates the probability *p* of the occurrence of the outcome under consideration and can be mathematically represented as follows (1):

(1) $$p=\frac{1}{1+e^{-\left(a_{0}+a_{1}x_{1}+a_{2}x_{2}+\cdots +a_{n}x_{n}\right)}}$$

where:

- *p* represents the probability of a given outcome, which ranges from 0 to 1.
- *x*_1_, *x*_2_, …, *x_n_* denote the values of the independent variables (risk factors) that are measured on nominal, ordinal, or quantitative scales.
- *a*_0_ signifies the intercept term of the model.
- *a*_1_, *a*_2_, …, *a_n_* are the regression coefficients corresponding to each independent variable.
- *e* denotes the base of the natural logarithm.

The stepwise forward selection method was utilized for the selection of independent variables, employing the Wald statistic as the criterion for exclusion. This methodology ensured that only statistically significant predictors were included in the final model. The overall statistical significance of the resulting model was assessed through the χ^2^ test.

To quantify the explanatory power of the logistic regression model, the Nagelkerke R^2^ was employed. This metric reflects the proportion of variance in the dependent variable that can be accounted for by the independent variables incorporated into the model. An elevated Nagelkerke R^2^ value indicates a superior fit of the model to the data. In order to evaluate the diagnostic significance of quantitative variables in predicting the outcome, Receiver Operating Characteristic (ROC) curve analysis was conducted. This approach facilitated the determination of the optimal threshold for classifying patients according to their risk levels, thereby achieving the best balance between sensitivity (true positive rate) and specificity (true negative rate).

The quality of the prognostic model was analyzed based on the area under the ROC curve (AUC), along with its standard error and 95% confidence interval (CI). Furthermore, the level of statistical significance (*p*-value) was reported to substantiate the robustness of the model, with the threshold for statistical significance set at *p* ≤ 0.05. It is noteworthy that missing values accounted for less than 1%.

- *Ethical Approval*: This study was approved by the local Ethics Committee at the “Scientific Center of Pediatrics and Pediatric Surgery” on 13 April 2022 (reference number: 2). We obtained informed consent from all participants’ authorized representatives before enrollment.
- *Trial registration:* The protocol was registered on ClinicalTrials.gov (NCT06094998) on 17 October 2023.

## 3. Results

The primary cohort comprised 120 children conceived by ART, while the control cohort included 132 children conceived spontaneously. The groups were comparable in terms of sex distribution: the primary cohort consisted of 56.7% boys and 43.3% girls, whereas the control cohort included 56.8% boys and 43.2% girls. As expected, parental age was significantly higher in the ART group compared with NC controls (median maternal age 34 vs. 28 years; paternal age 36 vs. 30 years, *p* < 0.001). Parity was higher in the NC group (median parity: ART 0 [IQR 0–1] vs. NC 1 [IQR 0–2], *p* < 0.001).

### 3.1. Prevalence of Nervous System Pathologies in Children Conceived by ART Compared to Naturally Conceived Children

We compared the frequency of nervous system pathologies, as well as various conditions that have the potential to affect the nervous system, in children, depending on their mode of conception (Table 1).

Consistent with the findings, the incidence of immaturity of brain structures detected by NSG was statistically significantly higher in the main group (*p* = 0.027). The odds of immaturity of brain structures of children born after ART were 2.75 times higher than those conceived spontaneously (95% CI: 1.09–6.93). When comparing the incidence of other pathologic conditions in children of both groups, there were no statistically significant differences.

### 3.2. Analysis of the Impact of ART and Other Risk Factors on the Development of Nervous System Pathologies and Cognitive Disorders in ART-Conceived Children

In light of the findings obtained, we conducted a comparative analysis of the frequency with which various factors influence the immaturity of brain structures, as determined by the NSG, across the studied groups. Table 2 summarizes associations across the full study sample (*n* = 252; ART + NC combined) and is not stratified by conception mode. Table 3 presents the analogous analysis restricted to ART-conceived children (*n* = 120).

Consistent with the findings, immaturity of brain structures diagnosed by the NSG was statistically significantly higher in the group of children from multiple pregnancies (OR = 5.48; 95% CI: 2.25–13.34), born prematurely (OR = 20.47; 95% CI: 7.63–54.94), with low birth weight (OR = 17.19; 95% CI: 46.58–44.91), with intrauterine pneumonia (OR = 10.69; 95% CI: 3.52–32.47), with respiratory distress syndrome (RDS) (OR = 10.68; 95% CI: 3.9–29.25), with neonatal asphyxia (OR = 8.19; 95% CI: 2.97–22.58), with pneumopathy (OR = 8.81; 95% CI: 2.54–30.57), with IUR (OR = 7.83; 95% CI: 2.03–30.16), with pathologic hyperbilirubinemia in the newborn period (OR = 2.84; 95% CI: 1.19–6.77), and with congenital heart disease (OR = 3.99; 95% CI: 1.55–10.29).

After accounting for factors such as multiple pregnancies, preterm birth, low birth weight, RDS, IUR, and congenital heart defects using binary logistic regression, the statistical significance of using ART disappeared. This indicates that the association between ART use and the development of immature brain structures is largely influenced by factors such as multiple pregnancies, preterm birth, and low birth weight.

In the logistic regression analysis, when ART was included in the model alongside significant risk factors, the use of ART itself was found to be statistically insignificant (*p* = 0.690; OR = 1.33; 95% CI: 0.33–5.36). This result further supports the conclusion that ART does not have an independent effect on the development of brain structure immaturity.

We further analyzed the influence of risk factors on the development of immaturity of brain structures in the cohort of children conceived by ART (Table 3).

Based on the collected data, the immaturity of brain structures diagnosed by NSG is statistically significantly higher among ART-conceived children. This increase is particularly notable in several specific groups: those born from multiple pregnancies (OR = 3.98; 95% CI: 1.29–12.34), those whose mothers received progesterone during pregnancy (OR = 3.56; 95% CI: 1.13–11.25), those born prematurely (OR = 22.18; 95% CI: 5.7–86.29), and those with low birth weight (OR = 14.14; 95% CI: 4.27–46.85). Additional contributing factors include intrauterine pneumonia (OR = 11.36; 95% CI: 2.65–48.68), RDS (OR = 8.31; 95% CI: 2.34–29.6), neonatal asphyxia (OR = 8.33; 95% CI: 1.84–37.71), pneumopathy (OR = 5.77; 95% CI: 1.16–28.71), and fetal growth restriction (OR = 5.77; 95% CI: 1.16–28.71). No statistically significant differences were observed when comparing the frequency of other risk factors.

### 3.3. Development of a Prediction Model for Determining the Probability of Immaturity of Brain Structures Detected by NSG in ART-Conceived Children

We developed a prognostic model to assess the probability of immaturity in brain structures detected by NSG in children conceived via ART, based on anamnestic factors using binary logistic regression. The observed relationship is described by Equation (2):

*p* = 1/(1 + e^(−) (z)^) × 100% z = −3.89 + 3.29 × X_PL_+ 1.7 × X_P_ (2)

where *p* is the probability of immaturity of brain structures detected by NSG in ART-conceived children (%), X_PL_ preterm labor (0—absence, 1—presence), X_P_ progesterone intake before pregnancy (0—absence, 1—presence).

The regression model obtained is statistically significant (*p* < 0.001). Based on the Nigelkerk coefficient of determination, 42.8% of the variance of the probability of detecting immaturity of brain structures detected by NSG in ART-conceived children is determined by the factors included in the model (2).

Based on the values of the regression coefficients, preterm birth and progesterone intake before pregnancy had a direct relationship with the probability of detecting immaturity of brain structures by NSG in ART-conceived children. The characteristics of each factor are presented in Table 4.

**Figure 1:**
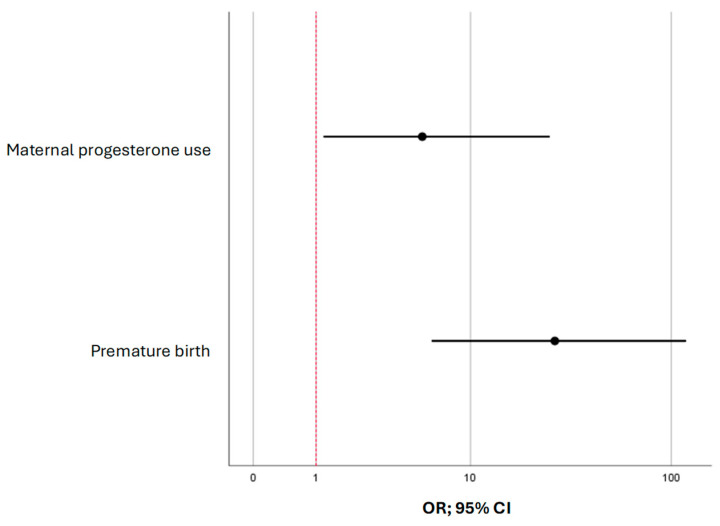
The forest plot shows odds ratios (ORs) with 95% confidence intervals (CIs) for the predictors of immature brain structures visualized by neurosonography in children conceived through assisted reproductive technologies.

Figure 1 compares the values of the adjusted odds ratios with 95% CIs for the studied factors included in model (2).

**Figure 2:**
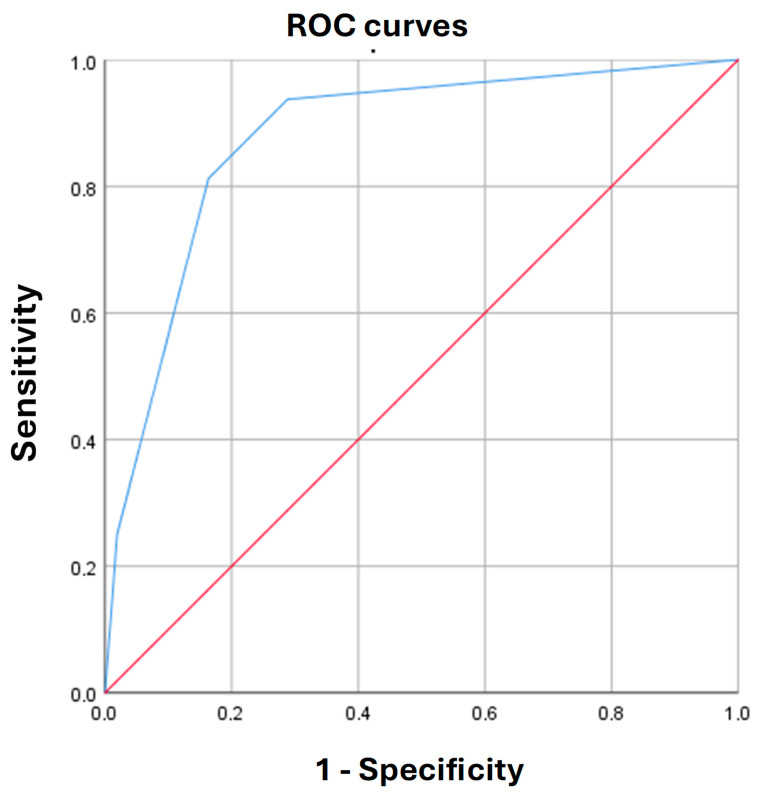
Receiver operating characteristic (ROC) curve characterizing the dependence of immature brain structures, visualized by neurosonography, in infants conceived by assisted reproductive technologies, on the values of the predictive (*p*) function (2).

The cutoff value of the logistic function *p* was determined using the ROC curve analysis. The resulting curve is shown in Figure 2.

The area under the ROC-curve was 0.88 ± 0.05 (95% CI: 0.79–0.97). The threshold value of the logistic function (2) at the cutoff point was 6%. *p* values greater than or equal to 6% indicated a high risk of detecting immaturity of brain structures by NSG in ART-conceived children, and *p* values < 6% defined a low risk of detecting immaturity of brain structures by NSG in ART-conceived children. The sensitivity and specificity of this model (2) at this threshold were 93.8% and 71.2%, respectively.

## 4. Discussion

Our study shows that ART-conceived children have a higher likelihood of immaturity of brain structures on NSG. We found no significant differences between the groups in other neurological conditions. This finding is consistent with several studies demonstrating a higher risk of white matter damage in preterm infants born after ART [4,27]. However, our multivariate analysis showed that ART itself is not an independent risk factor. The observed differences are primarily due to perinatal complications, including multiple pregnancies, preterm birth, low birth weight, asphyxia, and intrauterine infections [28,29]. These conclusions align with large cohort studies, where the increased risk of neurological disorders in ART-conceived children diminishes after accounting for factors like prematurity and multiple births [6,7]. In particular, gestational age and prematurity play a central role in shaping neurodevelopmental outcomes. In our analysis, prematurity and low birth weight—both directly linked to gestational maturity—were systematically included among the perinatal risk factors and were shown to have a significant effect on brain immaturity. This suggests that the observed differences in NSG findings are mediated primarily by gestational factors rather than ART itself. While subgroup statistical analysis by gestational week was not feasible due to the limited cohort size, the prognostic model confirms that prematurity is a key predictor of brain immaturity, supporting the robustness of our conclusions.

Our results contribute to the ongoing debate on long-term neurodevelopmental outcomes after ART. For example, Levin et al. reported increased neurological morbidity and ADHD in ART offspring [4], and Hansen et al. found an increased risk of intellectual disability in singleton births [8]. In contrast, other large-scale studies found that these risks are attenuated or absent after adjusting for prematurity or multiple gestations [6,7,11]. Some publications even suggest that ART children perform as well as or better than NC peers academically [11,12,13]. These conflicting results highlight the need to consider confounding perinatal factors rather than attributing risks solely to ART.

The prognostic model we developed showed that preterm birth and progesterone use in the pregravid period statistically significantly increased the probability of detecting immaturity of brain structures. These results are consistent with the findings of Bespalova et al. (2021) [30], where progestagen use in the management of multiple pregnancies after ART was associated with alterations in perinatal and neurological outcomes. However, the mechanisms of the effect of hormonal support on neurodevelopment remain poorly understood and require further analysis. Epigenetic alterations and environmental influences during IVF, culture conditions, and gamete or embryo cryopreservation have also been proposed as potential mechanisms influencing neurodevelopment [14,15,16].

Notably, we found no significant differences in the incidence of cognitive impairment, ADHD, psychiatric disorders, or CP between groups. This confirms other large studies that found no significant increase in long-term mental health risks for ART children [9,10]. Moreover, several studies indicate that the cognitive development and academic performance of these children are comparable to, or even surpass, that of their NC peers [11,12]. Wang et al. (2022) similarly concluded that ART-conceived children do not have a higher risk for poor mental health in adolescence, except for a slight increase in obsessive–compulsive disorder that may be related to parental factors [9].

It is also important to note the reported associations of ART with adverse perinatal outcomes, such as hypoxic–ischemic brain damage, hyperbilirubinemia, and neonatal hypoglycemia [3,25,31,32]. Although survival rates of preterm infants have improved, largely due to single embryo transfer policies [17,18,19,20,21,22,23,24,25,26], prematurity and its complications remain important mediators of neurological risk. The finding of elevated risks of cerebral white matter damage in preterm ART infants on MRI [27] further underscores the role of prematurity.

Taken together, our findings support the view that ART itself is not a direct independent risk factor for impaired neurodevelopment but rather interacts with perinatal factors that are already known to influence child outcomes.

**Limitations of the study.** The study has several limitations. First, the relatively small sample (120 children after ART and 132 in the control group) reduces statistical power and limits the ability to detect rare outcomes. Second, the one-stage observational design does not allow assessment of long-term cognitive and psychoemotional outcomes. Third, some of the data were obtained from medical history, which may have led to informational bias. Finally, the lack of stratification by type of ART (IVF, ICSI, fresh or cryopreserved embryo transfer) limits the interpretation of the results.

**Prospects for further research.** Future research should involve large prospective cohort studies that observe child development over the long term, focusing on cognitive, psycho-emotional, and social aspects. Notably, studies should examine epigenetic mechanisms, the impact of hormonal support during pregnancy, and how maternal somatic and endocrine diseases influence the development of the child’s nervous system. An important area of focus is developing algorithms for the early identification of at-risk groups and implementing prevention and rehabilitation programs. These initiatives aim to minimize the negative effects on the neuropsychological health of ART-conceived children.

## 5. Conclusions

The results of the study showed that the immaturity of brain structures according to NSG is more often detected in ART-conceived children. However, this issue is mostly linked to perinatal complications—such as multiple pregnancies, premature birth, low birth weight, asphyxia, and intrauterine infections—rather than the use of ART itself.

## Figures and Tables

**Table 1:** Comparison of the frequency of pathological conditions affecting the nervous system identified from medical history in the studied groups.

Pathological Conditions	Study Groups				*p*	OR; 95% CI
	ART (*n* = 120)		NC (*n* = 132)			
	Abs.	%	Abs.	%		
Intrauterine growth retardation	7	5.8	3	2.3	0.200	2.66; 0.67–10.54
Presence of birth trauma	6	5	5	3.8	0.761	1.34; 0.4–4.5
Asphyxia of the newborn	8	6.7	14	10.6	0.269	0.6; 0.24–1.5
Nervous system damage	17	14.2	12	9.1	0.207	1.65; 0.75–3.62
Immaturity of brain structures on neurosonography	16	13.3	7	5.3	0.027 *	2.75; 1.09–6.93
Pathologic hyperbilirubinemias of the newborn period	43	35.8	42	31.8	0.501	1.2; 0.7–2.02
Congenital malformations of the central nervous system (neural tube defects)	0	0	3	2.3	0.217	0.15; 0.0–3.0
Cerebral palsy	0	0	1	0.8	0.537	0.36; 0.01–9.02
Cognitive impairments	3	2.5	7	5.3	0.340	0.46; 0.12–1.81
Attention deficit hyperactivity disorder	6	5	8	6.1	0.788	0.82; 0.28–2.42
Autism spectrum disorders	3	2.5	2	1.5	0.671	1.67; 0.27–10.15
Mental disorders	1	0.8	2	1.5	1.000	0.55; 0.49–6.1

* differences are statistically significant (*p* < 0.05). Data are expressed in *n* (%). *p* values were determined using Chi-square or Fisher’s exact criterion for categorical data. Abbreviations: ART—children conceived by ART; NC—naturally conceived children; OR—odds ratio; CI—onfidence interval.

**Table 2:** Comparison of how various factors influence the likelihood of brain-structure immaturity in the combined cohort (*n* = 252; both children conceived through assisted reproductive technologies and those conceived naturally).

Risk Factors	Presence of Immaturity of Brain Structures on Neurosonography				*p*	OR; 95% CI	*p* **	aOR; 95% CI
	Presence (*n* = 23)		Absence (*n* = 229)					
	Abs.	%	Abs.	%				
Multiple pregnancy	12	52.2	38	16.6	<0.001 *	5.48; 2.25–13.34	0.736	1.22; 0.38–3.94
Abortion history	6	26.1	40	17.5	0.393	1.67; 0.62–4.49	0.015	3.38; 1.27–9.03
Maternal obesity	1	4.3	16	7	1.000	0.61; 0.08–4.78	0.032	2.75; 1.09–6.94
Thyroid diseases	3	13	58	25.3	0.305	0.44; 0.13–1.54	0.039	2.65; 1.05–6.72
Chronic pyelonephritis	9	39.1	58	25.3	0.213	1.9; 0.78–4.61	0.016	3.22; 1.24–8.35
Threat of abortion	10	43.5	73	31.9	0.256	1.64; 0.69–3.92	0.031	2.78; 1.1–7.02
Preterm labor	16	69.6	23	10	<0.001 *	20.47; 7.63–54.94	0.688	1.25; 0.43–3.66
Low birth weight	14	60.9	19	8.3	<0.001 *	17.19; 6.58–44.91	0.435	1.51; 0.54–4.28
Intrauterine pneumonia	7	30.4	9	3.9	<0.001 *	10.69; 3.52–32.47	0.042	2.74; 1.04–7.22
Respiratory distress syndrome	9	39.1	13	5.7	<0.001 *	10.68; 3.9–29.25	0.057	2.58; 0.97–6.82
Asphyxia of the newborn	8	34.8	14	6.1	<0.001 *	8.19; 2.97–22.58	0.011	3.78; 1.36–10.51
Pneumopathy (pulmonary atelectasis. hyaline membrane disease. edematous hemorrhagic syndrome)	5	21.7	7	3.1	0.002 *	8.81; 2.54–30.57	0.042	2.68; 1.04–6.94
Intrauterine growth retardation	4	17.4	6	2.6	0.008 *	7.83; 2.03–30.16	0.056	2.51; 0.98–6.44
Pathologic hyperbilirubinemia	13	56.5	72	31.4	0.020 *	2.84; 1.19–6.77	0.038	2.69; 1.06–6.84
Congenital heart defects	8	34.8	27	11.8	0.007 *	3.99; 1.55–10.29	0.064	2.43; 0.95–6.24

* Indicates statistical significance at *p* < 0.05. ** Estimates of the effect of ART on the frequency of immaturity of brain structures, adjusted for the effect of the corresponding factor. Data are expressed in *n* (%). *p* values were determined using Chi-square or Fisher’s exact test for categorical data. Abbreviations: OR—odds ratio; CI—confidence interval; aOR—adjusted odds ratio.

**Table 3:** Comparison of the frequency of influence of various factors on the probability of the development of immaturity of brain structures in children conceived by assisted reproductive technologies (ART).

Risk Factors		Presence of Immaturity of Brain Structures in ART-Conceived Children				*p*	OR; 95% CI
		Presence (*n* = 16)		Absence (*n* = 104)			
		Abs.	%	Abs.	%		
Multiple pregnancy		11	68.8	37	35.6	0.015 *	3.98; 1.29–12.34
Abortion history		3	18.8	8	7.7	0.164	2.77; 0.65–11.78
Thyroid diseases		0	0	25	24	0.105	0.09; 0.01–1.63
Chronic pyelonephritis		6	37.5	17	16.3	0.080	3.07; 0.98–9.58
Threatened miscarriage		8	50	31	29.8	0.151	2.36; 0.81–6.84
Preterm labor		13	81.3	17	16.3	<0.001 *	22.18; 5.7–86.29
Intracytoplasmic sperm injection		8	50	65	62.5	0.413	0.6; 0.21–1.73
Frozen Embryo Transfer		10	62.5	81	77.9	0.212	0.47; 0.16–1.44
Progesterone intake during pregnancy		6	37.5	15	14.4	0.035 *	3.56; 1.13–11.25
Low birth weight		11	68.8	14	13.5	<0.001 *	14.14; 4.27–46.85
Intrauterine pneumonia		5	31.3	4	3.8	0.002 *	11.36; 2.65–48.68
Respiratory distress syndrome		6	37.5	7	6.7	0.002 *	8.31; 2.34–29.6
Asphyxia of the newborn		4	25	4	3.8	0.011 *	8.33; 1.84–37.71
Pneumopathy (pulmonary atelectasis. hyaline membrane disease. edematous hemorrhagic syndrome)		3	18.8	4	3.8	0.049 *	5.77; 1.16–28.71
Fetal growth restriction	3	18.8		3	2.3	0.049 *	5.77; 1.16–28.71
Pathologic hyperbilirubinemia	9	56.3		34	32.7	0.093	2.65; 0.91–7.71
Congenital heart and vascular defects	5	31.3		17	16.3	0.170	2.33; 0.72–7.56

* Indicates statistical significance at *p* < 0.05. Data are expressed as *n* (%). *p* values were determined by using the Chi-square test or Fisher’s exact test for categorical data. Abbreviations: OR—odds ratio; CI—confidence interval.

**Table 4:** Relationship between predictors of model (2) and the probability of detecting immaturity of brain structures on the NSG in ART-conceived children.

Predictors	Unadjusted		Adjusted	
	COR; 95% CI	*p*	AOR; 95% CI	*p*
Preterm labor	22.18; 5.7–86.29	<0.001 *	26.92; 6.18–117.31	<0.001 *
Progesterone use before pregnancy	3.56; 1.13–11.25	0.035 *	5.46; 1.18–25.25	0.030 *

* Indicates statistical significance at *p* < 0.05. *p* values were determined by using the Chi-square test or Fisher’s exact test for categorical data. Abbreviations: COR—crude odds ratio; AOR—adjusted odds ratio; CI—confidence interval.

## Data Availability

The individual participant data that support the findings reported in this article, following deidentification, will be made available to other researchers who submit a methodologically sound proposal for an individual participant data meta-analysis. Proposals should be directed to ilmuratova.s@gmail.com. To obtain access to the data, requesters will be required to sign a data access agreement with the Scientific Center of Pediatric Surgery, as the intellectual property rights concerning the research results are held collectively, with the patent holder being the Scientific Center of Pediatrics and Pediatric Surgery. The study protocol will be publicly accessible on ClinicalTrials.gov (NCT06094998). There will be no endpoint specified.

## Abbreviations

The following abbreviations are used in this manuscript:

ART	Assisted Reproductive Technologies
IVF	In vitro fertilization
NC	Naturally conceived
ADHD	Attention deficit hyperactivity disorder
ASD	Autism spectrum disorders
CP	Cerebral palsy
ICSI	Intracytoplasmic sperm injection
OR	Odds ratio
CI	Confidence interval
ROC	Receiver Operating Characteristic
AUC	Area under the ROC curve
NSG	Neurosonography
IUR	Intrauterine growth retardation
RDS	Respiratory distress syndrome
